# Supplementary material for: The genome sequence of the Dotted Footman moth, Pelosia muscerda (Hufnagel, 1767)
Source: Wellcome Open Res. 2025 Feb 26;10:106. [Version 1] doi: 10.12688/wellcomeopenres.23766.1 (PMC11897693; doi:10.12688/wellcomeopenres.23766.1)
Supplement: Peer review history

# Reviewer report: Reviewer response for version 1

**DOI:** 10.21956/wellcomeopenres.26214.r120283

**Published:** 2025-04-04

**Authors:** Vlad Dinca

**Affiliations:** 1 University of Oulu, Oulu, Finland

In this data note, the authors report a chromosome-level genome assembly of a male *Pelosia muscerda* (Lepidoptera, Erebidae, Arctiinae). The mitochondrial genome has been assembled as well.

 Although I am not an expert in several of the methodological approaches used, as far as I can tell, the manuscript uses methodologies and pipelines that are fairly well-established through use by the Sanger Institute. Data quality seems good (e.g. BUSCO score of 98.6% and other parameters summarized in Table 2).

 I suggest including a bit of additional information in the Background section. For example, a few words about the general distribution of the species, its larval host plant(s), phenology, preferred habitat etc.

Are sufficient details of methods and materials provided to allow replication by others?

Yes

Is the rationale for creating the dataset(s) clearly described?

Yes

Are the datasets clearly presented in a useable and accessible format?

Yes

Are the protocols appropriate and is the work technically sound?

Yes

Reviewer Expertise:

Lepidoptera speciation, taxonomy and phylogeography, with a focus on Western Palearctic butterflies.

I confirm that I have read this submission and believe that I have an appropriate level of expertise to confirm that it is of an acceptable scientific standard.

# Reviewer report: Reviewer response for version 1

**DOI:** 10.21956/wellcomeopenres.26214.r119672

**Published:** 2025-03-13

**Authors:** Christophe Klopp

**Affiliations:** 1 INRAE, Castanet-Tolosan, France

The authors present the first chromosomal assembly of the Dotted Footman moth, *Pelosia muscerda.*

 The article is simple to read but the methods are a bit outdated. It is not clear from the material and methods if the authors have used the Hi-C data during the assembly but it seems that is was the case. This is good practice because it enables to build better haplotypes. 

 The authors have chosen to produce principal and alternative assemblies and not to scaffold the alternative which is as long as the principal. Producing scaffolded hap1 and hap2 would have been better for future analysis such as building a pangenome which could be of high interest in a species showing high heterozygosity.

Are sufficient details of methods and materials provided to allow replication by others?

Partly

Is the rationale for creating the dataset(s) clearly described?

Yes

Are the datasets clearly presented in a useable and accessible format?

Yes

Are the protocols appropriate and is the work technically sound?

Partly

Reviewer Expertise:

Bioinformatics, genome assembly and annotation

I confirm that I have read this submission and believe that I have an appropriate level of expertise to confirm that it is of an acceptable scientific standard, however I have significant reservations, as outlined above.

# Reviewer report: Reviewer response for version 1

**DOI:** 10.21956/wellcomeopenres.26214.r120277

**Published:** 2025-03-11

**Authors:** Arun Arumugaperumal

**Affiliations:** 1 Department of Biotechnology, Rajalakshmi Engineering College, Thandalam, Chennai, Tamilnadu, 602015, India

This is the first whole genome report of the Dotted Footman moth, *Pelosia muscerda*. The assembly reported here is of size 500.49 Mb and the mitogenome is of size 15.42 kb. The authors can shed some more light on how they identified misjoins. Did they use any software or script to compare sequence contigs? In Figure 1 caption there is no explanation of what is a and b labels in the figure. BUSCO analysis has shown that the genome is 98.6% complete. The report can be accepted for indexing.

Are sufficient details of methods and materials provided to allow replication by others?

Yes

Is the rationale for creating the dataset(s) clearly described?

Yes

Are the datasets clearly presented in a useable and accessible format?

Yes

Are the protocols appropriate and is the work technically sound?

Yes

Reviewer Expertise:

Bioinformatics, Genomics

I confirm that I have read this submission and believe that I have an appropriate level of expertise to confirm that it is of an acceptable scientific standard.

# Reviewer report: Reviewer response for version 1

**DOI:** 10.21956/wellcomeopenres.26214.r120285

**Published:** 2025-03-10

**Authors:** Bhagya C. Thimmappa

**Affiliations:** 1 US Department of agriculture, USDA Agricultural Research Service (Ringgold ID: 17123), New Jersey, Chatsworth, USA

In the article “The genome sequence of the Dotted Footman moth, *Pelosia muscerda *(Hufnagel, 1767)”, the authors report the nuclear and mitochondrial genome of the Dotted Footman moth. They performed sequencing using Pacific Biosciences single-molecule HiFi long reads and chromosome conformation Hi-C sequencing. The reported assembly is 500.49 Mb in length across 35 scaffolds, which is highly complete, with a BUSCO score of 98.6%.

**Major comments:**

1. Upon cross-checking NCBI taxonomy, *Pelosia muscerda* (Hufnagel, 1767) should be *Pelosia muscerda* (Hufnagel, 1766).
2. Background information is limited. What is the geographical distribution of *Pelosia muscerda*, its life cycle, and does it cause any issues for plants? In other words, more information is needed to highlight why the reported dataset is essential.

**Minor comments:**

1. More information on the RNA-Seq data files and when the annotation will be done would be helpful.
2. If possible, mitochondrial annotation would be helpful for further evolutionary studies.

Are sufficient details of methods and materials provided to allow replication by others?

Yes

Is the rationale for creating the dataset(s) clearly described?

Partly

Are the datasets clearly presented in a useable and accessible format?

Yes

Are the protocols appropriate and is the work technically sound?

Yes

Reviewer Expertise:

Genomics, Bioinformatics, multi-omics

I confirm that I have read this submission and believe that I have an appropriate level of expertise to confirm that it is of an acceptable scientific standard.
